# Supplementary material for: ROBO2 is a stroma suppressor gene in the pancreas and acts via TGF-β signalling
Source: Nat Commun. 2018 Nov 30;9:5083. doi: 10.1038/s41467-018-07497-z (PMC6269509; doi:10.1038/s41467-018-07497-z)
Supplement: Supplementary file 1 — Supplementary Information [file 41467_2018_7497_MOESM1_ESM.pdf]

ROBO2 is a stroma suppressor gene in the pancreas and acts via TGF-β signalling

Pinho et al.

SUPPLEMENTARY MATERIAL

SUPPLEMENTARY FIGURES

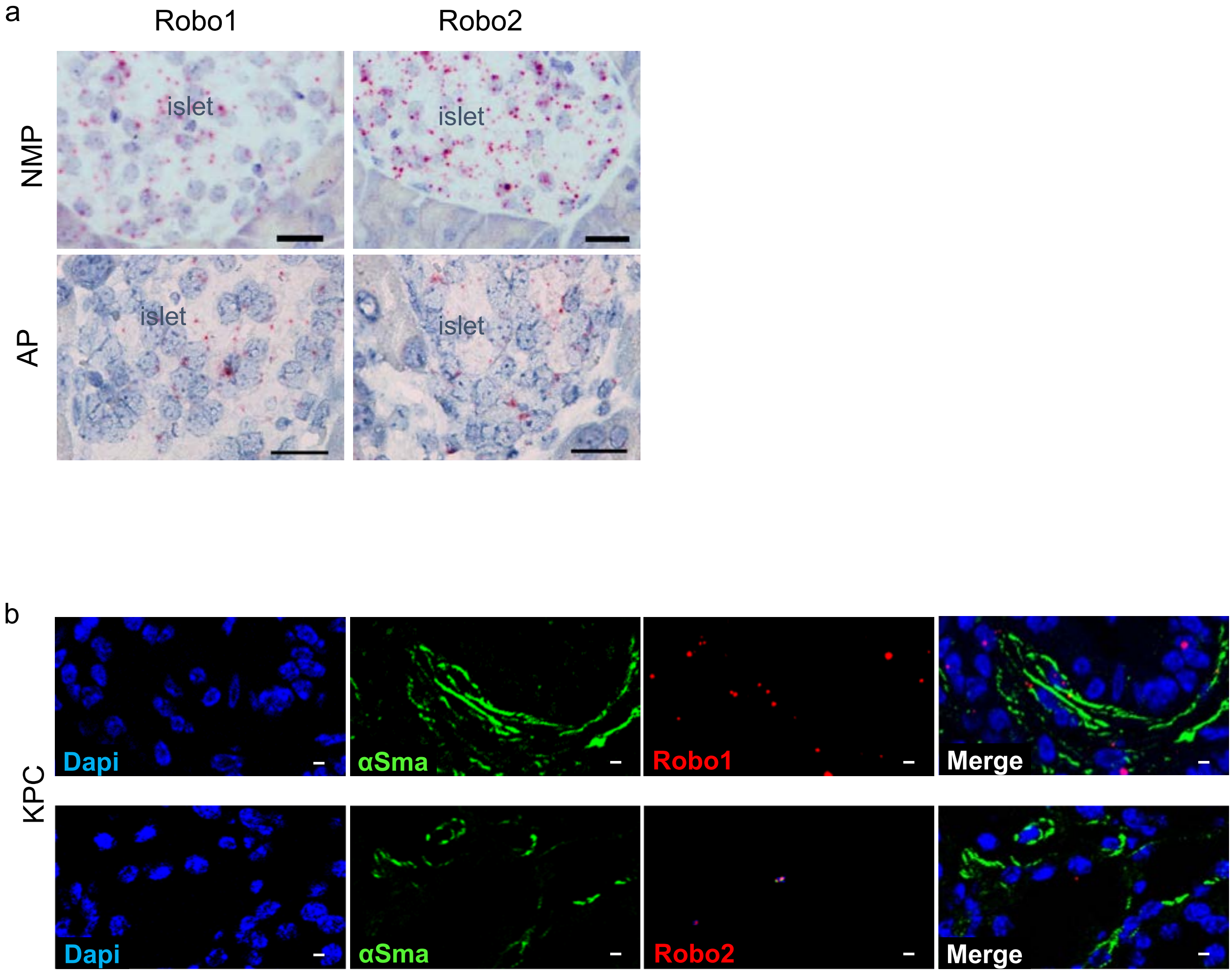

Supplementary Figure 1. RNA in situ hybridization of Robo1 and Robo2 in murine pancreatic tissue

a. Robo1/2 mRNA expression in islets of normal mouse pancreas (NMP) and acute pancreatitis (AP). RNA in situ hybridization (RISH) for Robo1 and Robo2. Note that dot intensity is not related to the amount of mRNA copies. Images are representative from 6 independent experiments Scale bars are 20µm.

b. RISH - immunofluorescence multiplexing of Robo1 or Robo2 with the mesenchymal marker α-Sma in PDAC (KPC model). Nuclei are stained with Dapi. Confocal pictures were acquired using 20x magnification with zoom 2. Images are representative from 3 independent experiments. Scale bars are 10µm.

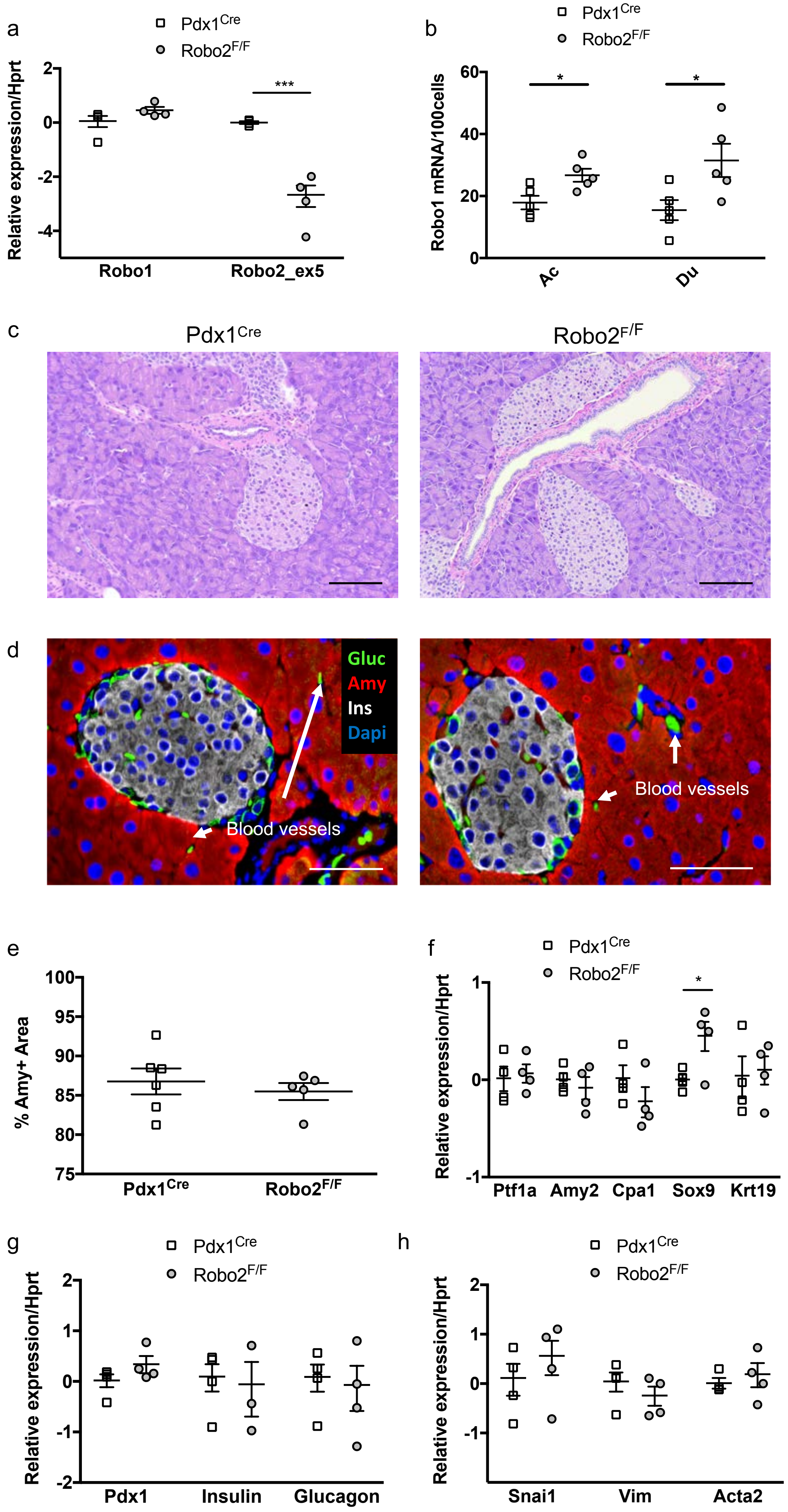

a
Pdx1Cre
Robo2F/F
Relative expression/Hprt
***
Robo1
Robo2_ex5
b
Robo1 mRNA/100cells
*
*
Ac
Du
c
Pdx1Cre
Robo2F/F
d
Gluc
Amy
Ins
Dapi
Blood vessels
Blood vessels
e
% Amy+ Area
Pdx1Cre
Robo2F/F
f
Relative expression/Hprt
*
Ptf1a
Amy2
Cpa1
Sox9
Krt19
g
Relative expression/Hprt
Pdx1
Insulin
Glucagon
h
Relative expression/Hprt
Snai1
Vim
Acta2

Supplementary Figure 2. Robo2$^{F/F}$ animals present normal pancreatic tissue histology and gene expression

a. mRNA expression of Robo receptors analysed by RT-qPCR in pancreatic tissue of 6 month-old Pdx1^{Cre} and Robo2$^{F/F}$ animals. All qPCR data is referred to housekeeping gene Hprt. All data is presented as Mean +/- SEM; N=4, Statistical analysis was performed using an unpaired t test with Welsh's correction; *P<0.05, **P<0.01, ***P<0.001.

b. Quantification of Robo1 RISH in NMP of Pdx1^{Cre} and Robo2$^{F/F}$ animals. Ac, Acini; Du, Ducts. Data is presented as Mean +/- SEM; N=5. Statistical analysis was performed using an unpaired t test with Welsh's correction; *P<0.05, **P<0.01, ***P<0.001.

c. Haematoxylin and eosin staining of pancreas sections of 6 month-old Pdx1^{Cre} and Robo2$^{F/F}$ animals. Scale bars are 50µm. Images acquired using 20x magnification**.**

d. Immunofluorescence staining for Insulin, Glucagon and Amylase showing no difference between Pdx1^{Cre} and Robo2$^{F/F}$. Blood shows unspecific staining in the green channel. Images are representative from 5 independent experiments. Scale bars are 50µm. Images acquired using 20x magnification.

e. Quantification of Amylase+ area/field analysed using Celleste. Data is presented as Mean +/- SEM; N≥5.

f-h. mRNA expression of exocrine (f), endocrine (g) and mesenchymal (h) cell markers analysed by RT-qPCR in pancreatic tissue of 6 month-old Pdx1^{Cre} and Robo2$^{F/F}$ animals. All qPCR data is referred to housekeeping gene Hprt. All data is presented as Mean +/- SEM; N=4, Statistical analysis was performed using an unpaired t test with Welsh's correction; *P<;0.05, **P<0.01, ***P<0.001.

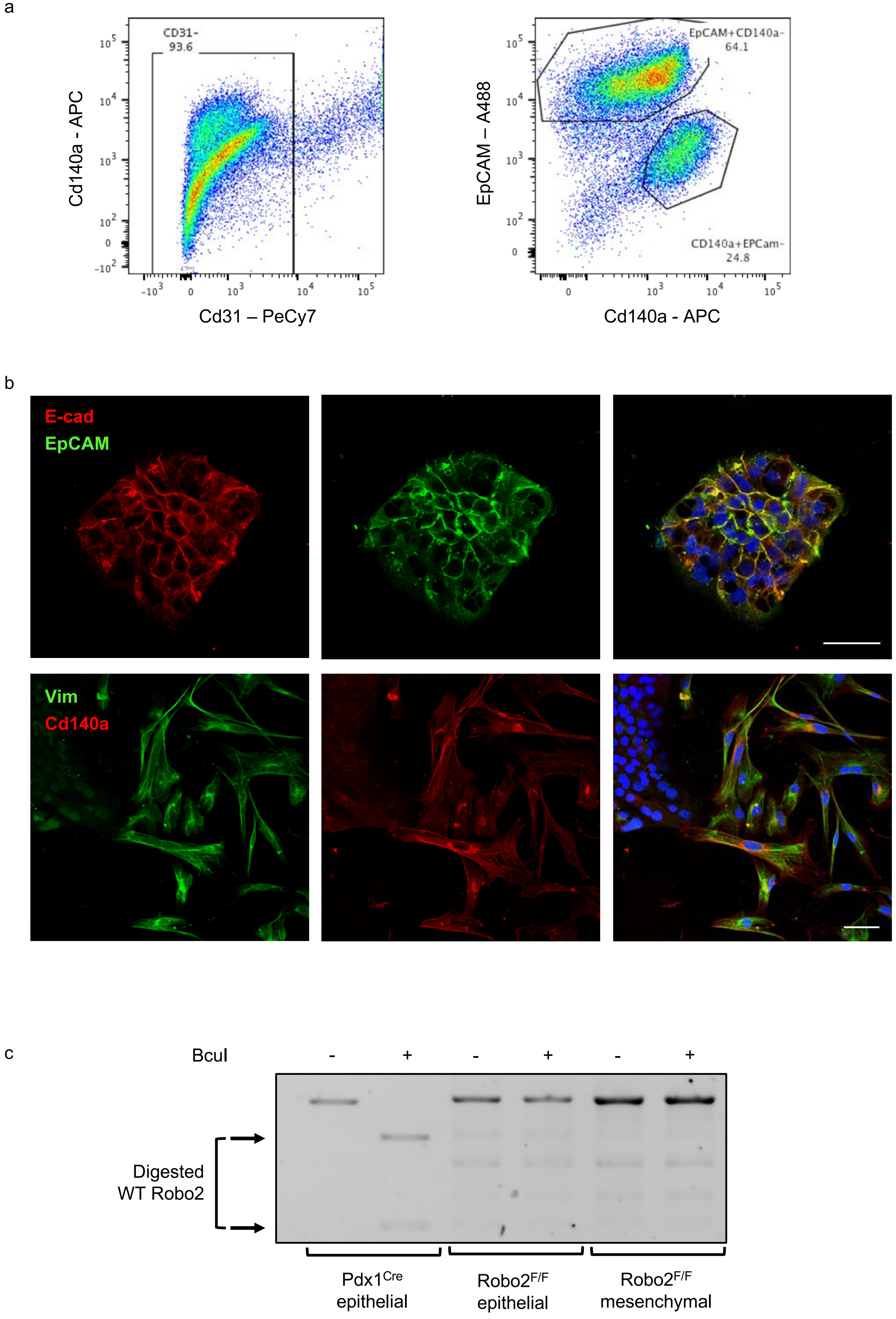
a
CD31-
93.6
Cd140a - APC
Cd31 – PeCy7
EpCAM+CD140a-
64.1
CD140a+EPCam-
24.8
EpCAM – A488
Cd140a - APC
b
E-cad
EpCAM
Vim
Cd140a
c
BcuI
-
+
-
+
-
+
Digested
WT Robo2
Pdx1Cre
epithelial
Robo2F/F
epithelial
Robo2F/F
mesenchymal

Supplementary Figure 3. FACS-sorting of Robo2$^{F/F}$ pancreatic exocrine cell cultures

a. Flow cytometry gating strategy utilized for FACS sorting of epithelial (EpCAM^{+}, Cd31^{-}, Cd140a^{-}) and mesenchymal (Cd140a^{+}, Cd31^{-}, EpCAM^{-}) cell populations in Day 8 Pdx1^{Cre} and Robo2$^{F/F}$ cultures.

b. Immunofluorescence of markers used for FACS-sorting of Robo2$^{F/F}$ pancreatic exocrine cell cultures. EpCAM co-localizes with E-cadherin in epithelial cells and Cd140a colocalizes with Vimentin in mesenchymal cells. Nuclei are stained with Dapi. Images are representative from 3 independent experiments. Confocal microscope images were acquired at 40x magnification. Scale bars represent 50 µm.

c. Genotyping of genomic DNA extracted from FACS-sorted Day 8 Pdx1^{Cre} and Robo2$^{F/F}$ cultures. Upon BcuI restriction the 1100bp amplicon is digested into a 750bp and 350bp amplicon in Pdx1^{Cre} controls whereas the 1100bp remains intact in Robo2$^{F/F}$ mice.

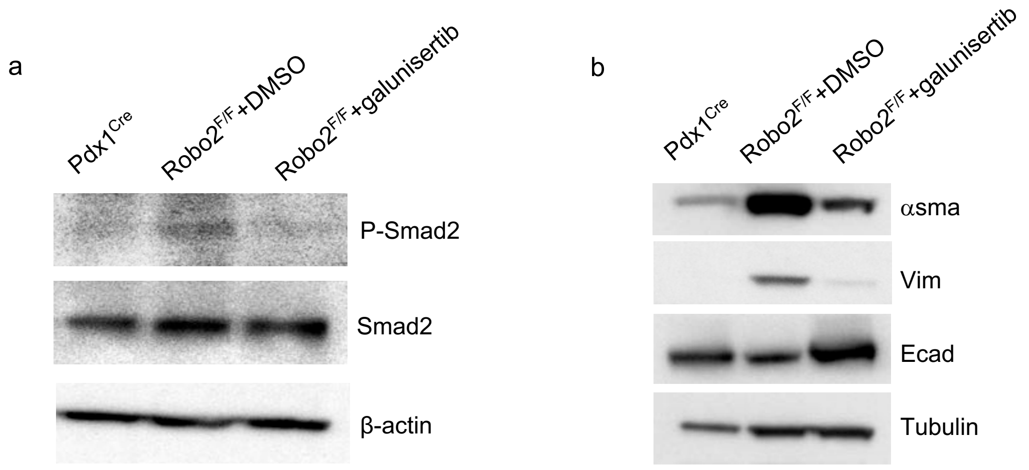

Supplementary Figure 4. Protein expression changes in Robo2$^{F/F}$ pancreatic cell cultures

a. Western blot (WB) analysis of P-Smad2 and Total Smad2 in D8 pancreatic cultures treated with galunisertib or DMSO vehicle.

b. Western blot (WB) analysis of the mesenchymal markers α-Sma and Vimentin and the epithelial marker E-cadherin in D8 pancreatic cultures treated with galunisertib or DMSO vehicle.

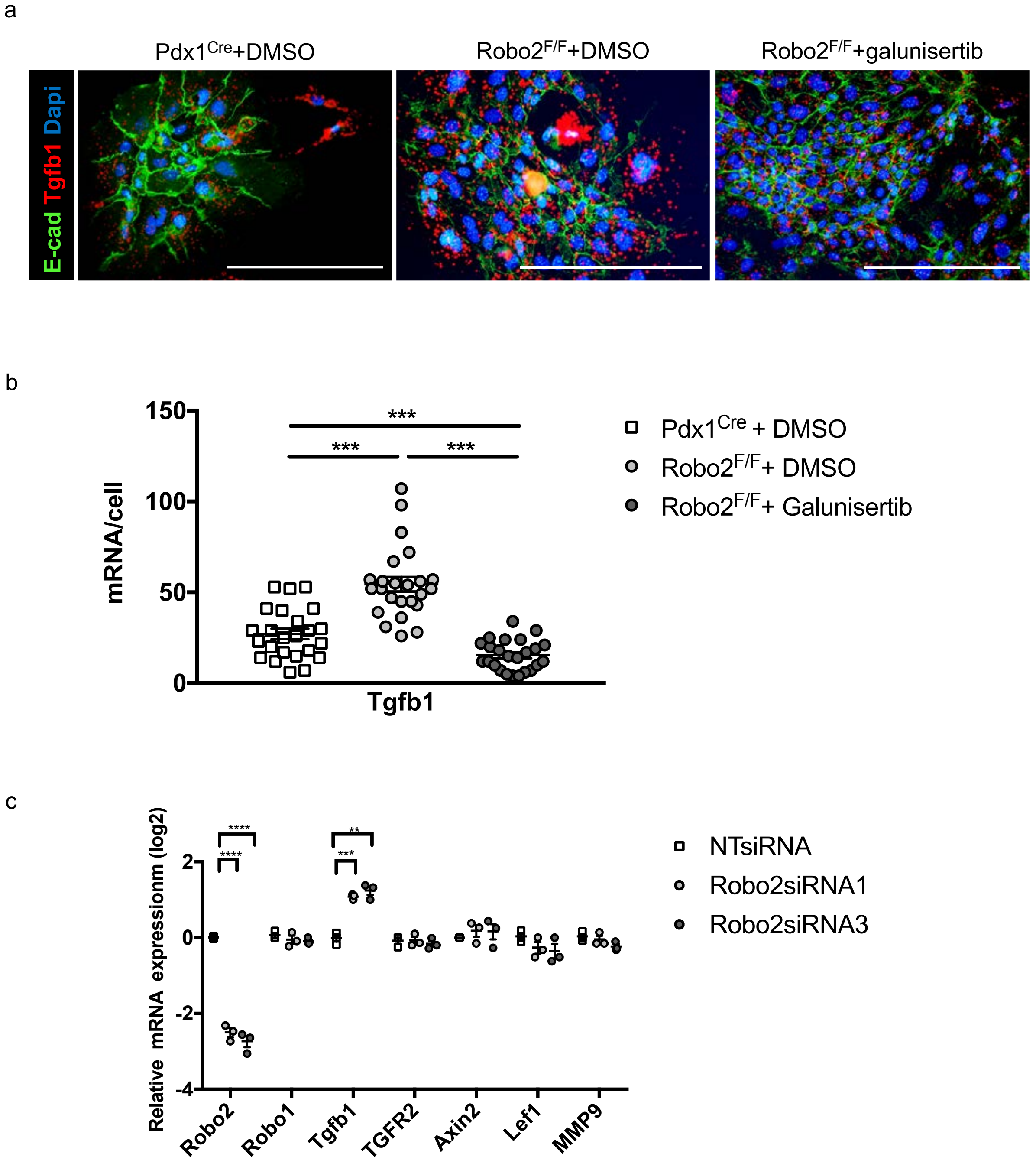

Supplementary Figure 5. Tgfβ1 upregulation in epithelial cells upon Robo2 knock down.

a. Images of RISH-immunofluorescence multiplexing for Tgfβ1 ligand mRNA and epithelial E-Cad in $Pdx1^{Cre}$ and $Robo2^{F/F}$ primary pancreatic cultures. Nuclei are stained with DAPI. Images acquired using 20x magnification. Images are representative from 3 independent experiments. Scale bars correspond to 200μm.

b. Quantification of Tgfβ1 mRNA in E-cad^{+} cells.

c. Panc1 cells were transfected with one of two different siRNAs and the analysis was done on day 4 after transfection. qRT-PCR analysis of Robo2, Robo1, TGF-β and Wnt target genes.

All data is presented as Mean+/- SEM, N≥3. Statistical analysis was performed using an unpaired t test with Welsh's correction; *P<0.05, **P<0.01, ***P<0.001 ****P=<0.0001.

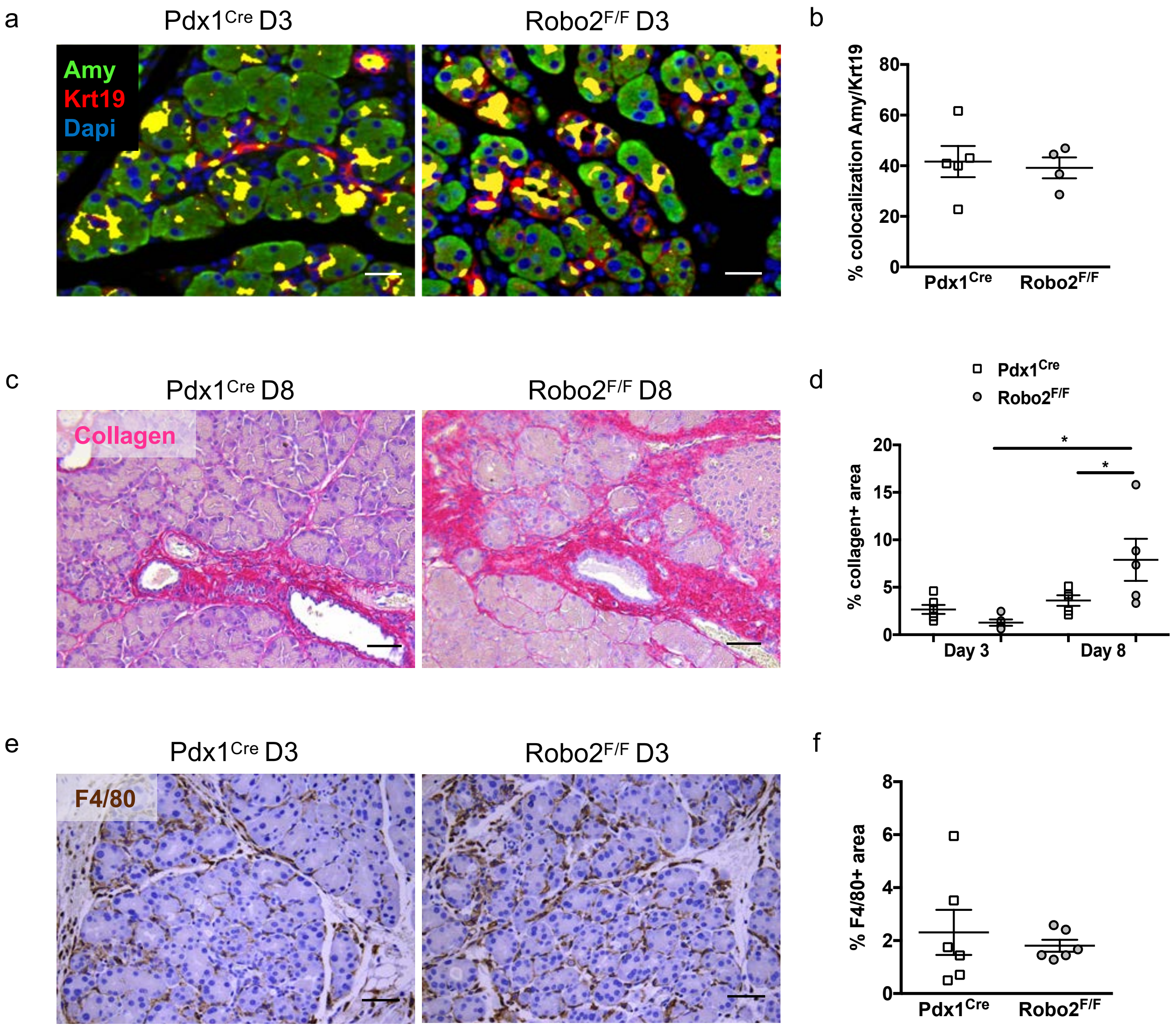

Supplementary Figure 6. Analysis of Robo2$^{F/F}$ pancreas after acute pancreatitis

a. Representative images of acinar-to-ductal metaplasia (ADM) quantification by co-localization of acinar marker, Amylase (Amy, green) and ductal marker, Keratin19 (Krt19, red) in Pdx1^{Cre} and Robo2$^{F/F}$ mice at D3 acute pancreatitis (AP).
b. Quantification of ADM in Pdx1^{Cre} and Robo2$^{F/F}$ mice at D3 AP.
c. Picrosirius red staining for collagen at D3 and D8 in Pdx1^{Cre} and Robo2$^{F/F}$ mice.
d. Quantification of picrosirius stained area at D3 and D8 in Pdx1^{Cre} and Robo2$^{F/F}$ mice using ImageJ.
e. Immunohistochemistry analysis of F4/80^{+} macrophages in pancreatic tissue at D3 AP in Pdx1^{Cre} and Robo2$^{F/F}$ mice.
f. Area quantification of F4/80^{+} macrophages in pancreatic tissue at D3 AP in Pdx1^{Cre} and Robo2$^{F/F}$ mice using ImageJ.
Scale bars correspond to 50μm. All All data is presented as Mean +/- SEM; N≥4. Statistical analysis was performed using an unpaired t test with Welsh's correction; *$P<0.05$.

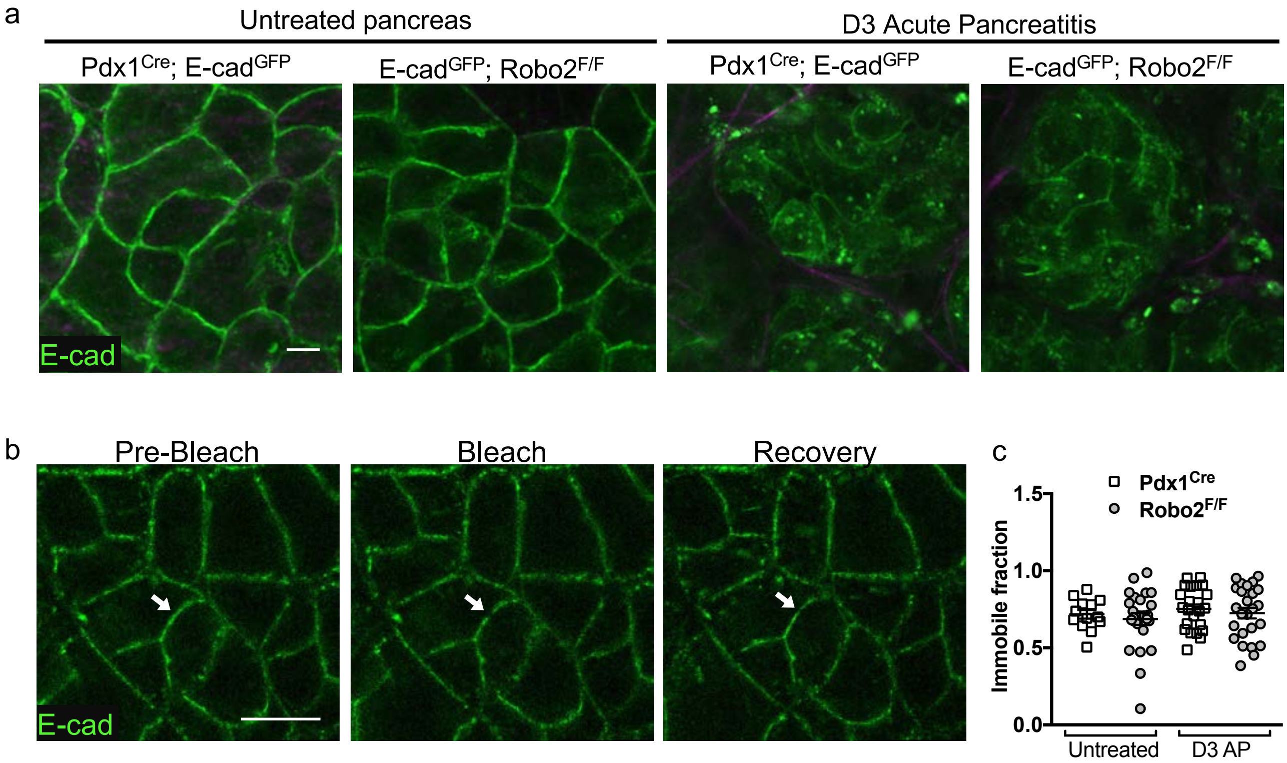

Supplementary Figure 7. FRAP analysis of E-cadherin in Robo2$^{F/F}$ animals

a. E-cad^{GFP} localization in live pancreas of E-cad^{GFP}; Pdx1^{Cre} and E-cad^{GFP}; Pdx1^{Cre}; Robo2$^{F/F}$ animals before and after AP (D3) using multiphoton microscopy. Scale bars correspond to 10μm.

b. Representative Fluorescence Recovery After Photobleach (FRAP) images in Robo2$^{F/F}$ untreated pancreas using confocal microscopy. Scale bars correspond to 25μm **c.** Quantification of immobile fraction after FRAP analysis. Data is presented as Mean +/- SEM; N animals/group ≥3.

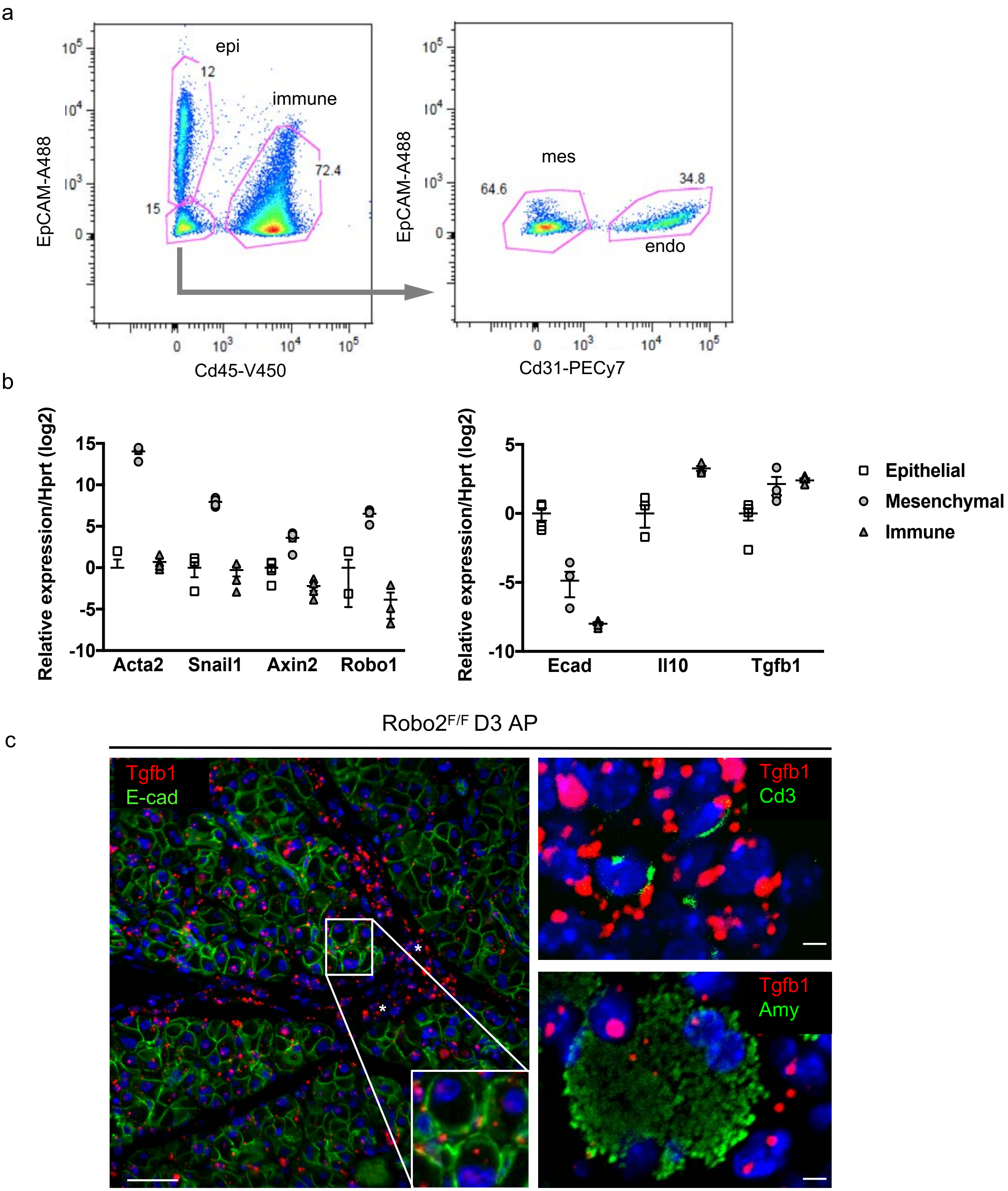

Supplementary Figure 8. Analysis of distinct cellular populations in Robo2$^{F/F}$ animals after AP

a. Example of gating strategy used for FACS sorting of different cell populations (EpCAM^{+} epithelial cells, Cd45+ immune cells, Cd31+ endothelial cells and triple negative mesenchymal cells) in total pancreatic tissue after acute pancreatitis.

b. Quantification of the different cell populations in Pdx1^{Cre} and Robo2$^{F/F}$ animals at D3 AP**.** Data presented as Mean +/- SEM, relative to the average of Pdx1^{Cre} controls; N=4-6; *P<0.05.

c. RISH-immunofluorescence multiplexing for Tgfβ1 ligand mRNA and epithelial (E-Cad, Amy) and immune markers (Cd3) in Robo2$^{F/F}$ pancreas at D3 AP. Images are representative from 3 independent experiments. Scale bars correspond to 100μm and 10μm for right panel and left panel respectively.

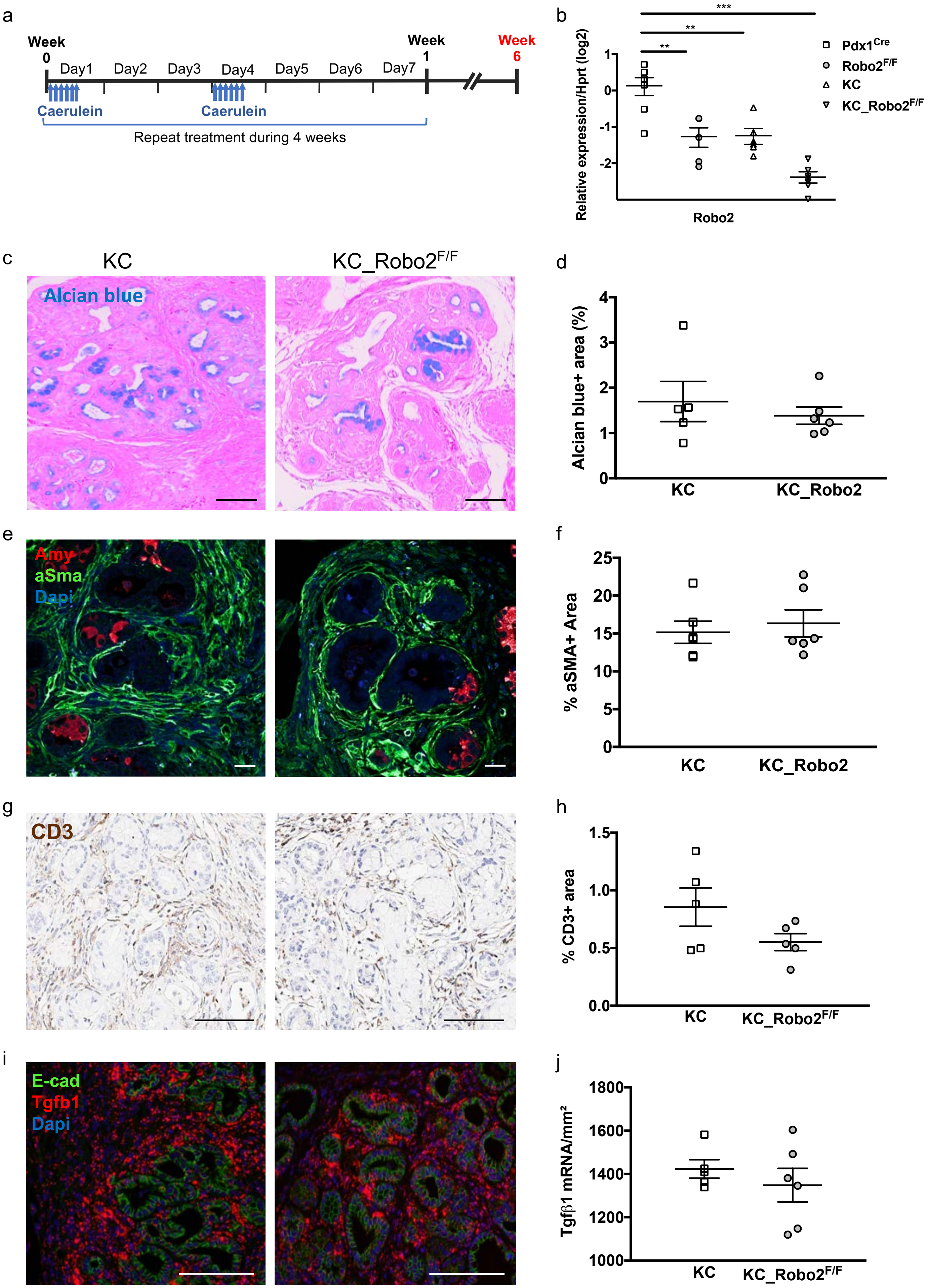
a
Week 0
Day1
Day2
Day3
Day4
Day5
Day6
Day7
Week 1
Week 6
Caerulein
Caerulein
Repeat treatment during 4 weeks
b
***
**
**
Relative expression/Hprt (log2)
Robo2
Pdx1Cre
Robo2F/F
KC
KC_Robo2F/F
c
KC
KC_Robo2F/F
Alcian blue
d
Alcian blue+ area (%)
KC
KC_Robo2
e
Amy
aSma
Dapi
f
% aSMA+ Area
KC
KC_Robo2
g
CD3
h
% CD3+ area
KC
KC_Robo2F/F
i
E-cad
Tgfb1
Dapi
j
Tgfβ1 mRNA/mm²
KC
KC_Robo2F/F

Supplementary Figure 9. Chronic pancreatitis in mice with concurrent loss of Robo2 and $Kras^{G12D}$

a. Chronic pancreatitis was induced in $Kras^{G12D}$;$Pdx1^{Cre}$ (KC) and $Kras^{G12D}$;$Pdx1^{Cre}$;$Robo2^{F/F}$ ($KC\_Robo2^{F/F}$) animals by continuous caerulein treatment during 4 weeks. Animals were sacrificed 2 weeks after termination of treatment (week 6), at 6 months of age.
b. Robo2 mRNA expression referred to housekeeping gene Hprt. Data are presented as Mean +/- SEM; N=4-5. Statistical test was performed using One-Way Anova and Turkey's multicomparisons test, **P<0.01, ***P<0.001.
c. Alcian blue staining of pancreas sections at week 6. Images were acquired using 10x magnification. Scale bars correspond to 200µm.
d. Quantification of alcian blue+ area/field, analyzed using ImageJ.
e. Representative images of α-Sma and Amy immunofluorescence in KC and $KC\_Robo2^{F/F}$ pancreata. Nuclei are stained with Dapi. Images were acquired using 40x magnification. Scale bars correspond to 25µm
f. Quantification of α-Sma+ area/field, analyzed using ImageJ.
g. Images of CD3 immunohistochemistry in KC and $KC\_Robo2^{F/F}$ pancreata. Images were acquired using 20x magnification. Images are representative from 5 independent experiments. Scale bars correspond to 100µm.
h. Quantification of CD3+ area/field, analyzed using Celleste.
i. Images of RISH-immunofluorescence multiplexing for Tgfβ1 ligand mRNA and epithelial E-Cad in KC and $KC\_Robo2^{F/F}$ pancreata. Images were acquired using 20x magnification. Images are representative from 5 independent experiments. Scale bars correspond to 100µm.
j. Quantification of total Tgfb1 mRNA in KC and $KC\_Robo2^{F/F}$ pancreata.
All Data are presented as Mean +/- SEM; N=5-6.

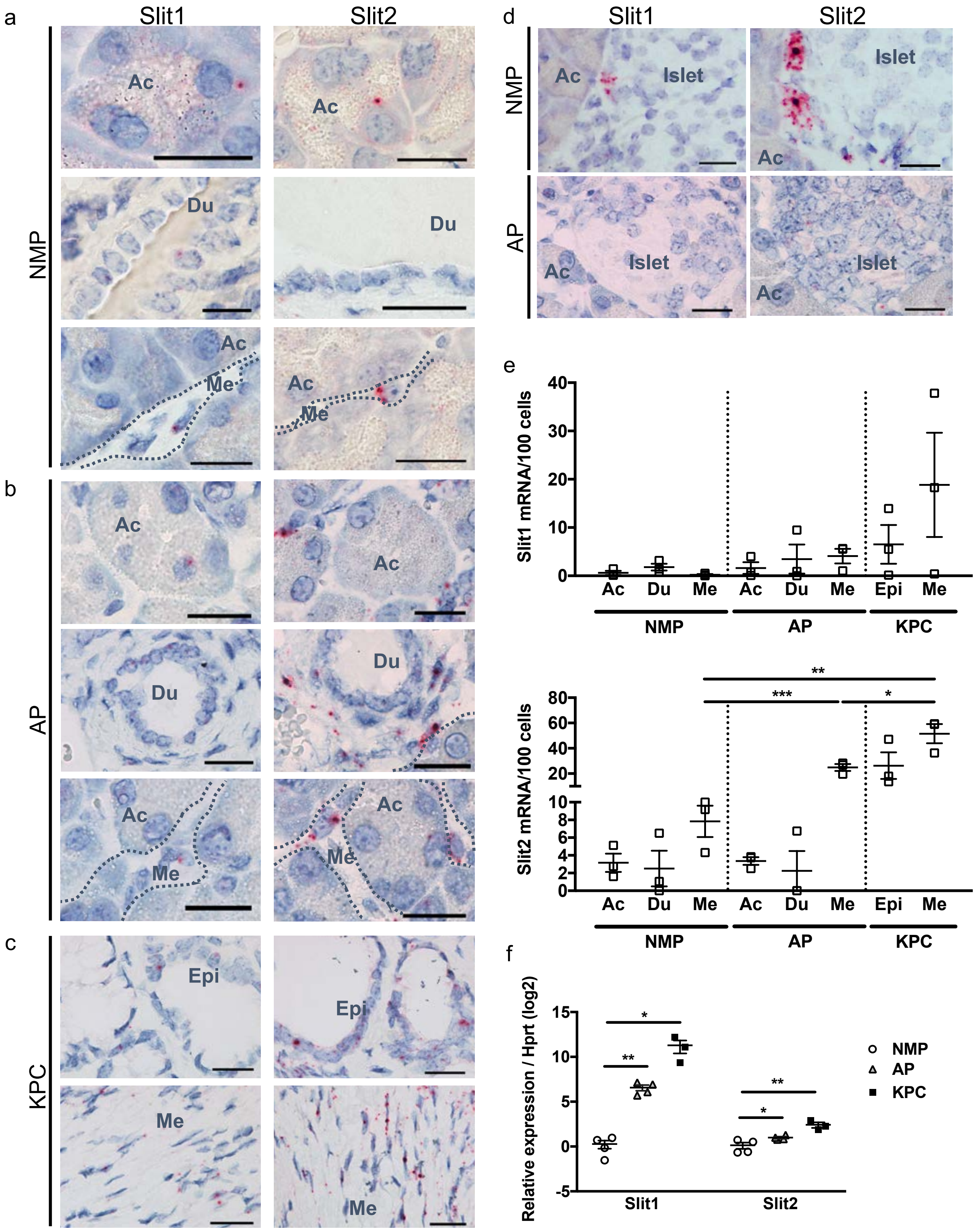

Supplementary Figure 10. Slit1-2 ligands show cell-type specific expression changes

a-d. Slit1 and Slit2 mRNA expression analyzed by RNA in situ hybridization (RISH) in **a.** normal mouse pancreas (NMP), **b.** acute pancreatitis (AP), **c.** PDAC (KPC model), **d.** islets of normal mouse pancreas and acute pancreatitis. Images are representative from 3 independent experiments. Scale bars correspond to 20µm. Dotted lines delineate histological compartments (Acini, Ac; Ducts, Du; Tumour epithelium, Epi; Mesenchymal cells, Me). **e.** Quantification of Slit1 and Slit2 RISH in sections of NMP, AP and KPC (Acini, Ac; Ducts, Du; Tumor epithelium, Epi; Mesenchymal cells, Me). Note that dot intensity is not related to the amount of mRNA copies. **f.** mRNA expression of Slit1 and Slit2 in NMP, AP and KPC analysed by RT-qPCR. Scale bars correspond to 20µm. All data is presented as Mean +/- SEM; N≥3. Statistical analysis was performed using an unpaired t test with Welsh's correction; *P<0.05, **P<0.01, ***P<0.001.

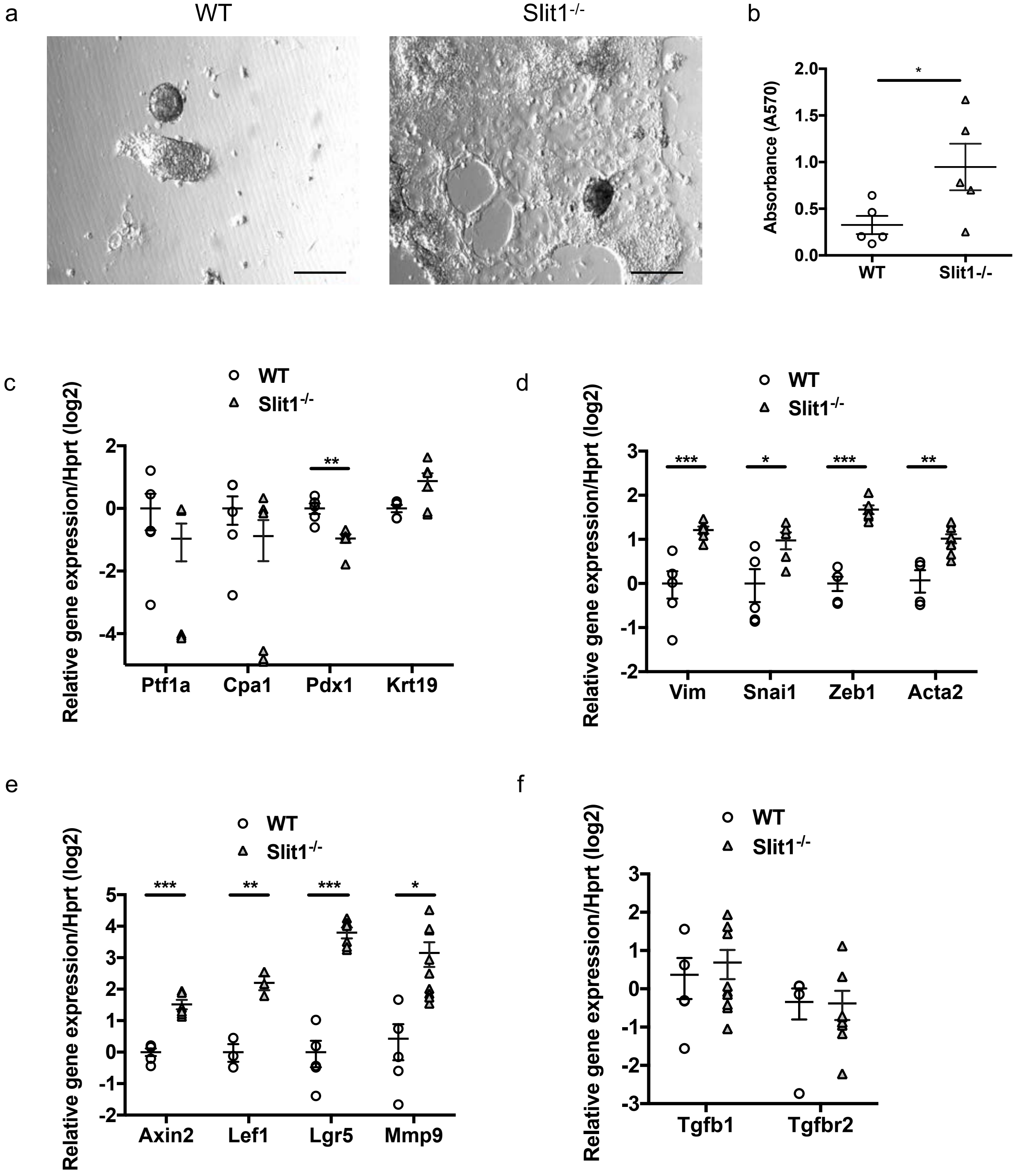

Supplementary Figure 11. Slit1$^{-/-}$ pancreatic exocrine cell cultures

a. Representative images of pancreatic exocrine cultures at day 8(D8). Scale bars correspond to 200µm
b. Quantification of Sulphorodamine B staining as a measurement of cell attachment at 48h of culture**.**
c-f. mRNA expression of epithelial (c) and mesenchymal (d) markers, Wnt targets (e) and TGF-β pathway genes (f) analysed by RT-qPCR in pancreatic exocrine cultures at D8.
All qPCR data is referred to housekeeping gene Hprt. Data presented as Mean +/- SEM; N=4-6, Statistical analysis was performed using an unpaired t test with Welsh's correction; *P<0.05, **P<0.01, ***P<0.001.

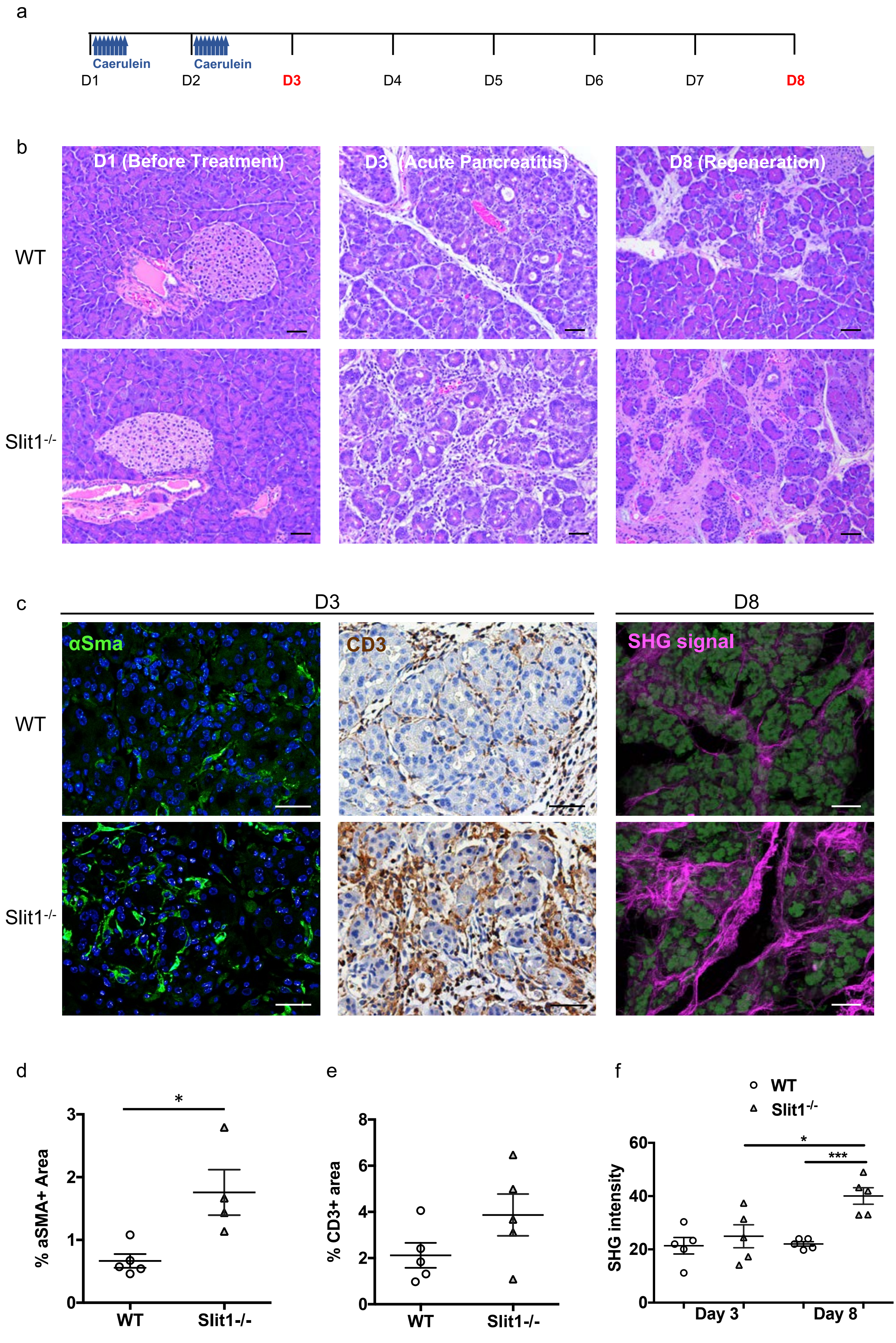
a
Caerulein
Caerulein
D1
D2
D3
D4
D5
D6
D7
D8
b
D1 (Before Treatment)
D3 (Acute Pancreatitis)
D8 (Regeneration)
WT
Slit1-/-
c
D3
D8
αSma
CD3
SHG signal
WT
Slit1-/-
d
e
f
% aSMA+ Area
WT
Slit1-/-
% CD3+ area
WT
Slit1-/-
WT
Slit1-/-
SHG intensity
Day 3
Day 8

Supplementary Figure 12. Slit1 ligand suppresses stromal remodelling in acute pancreatitis

a. Acute pancreatitis (AP) was induced in WT and Slit1$^{-/-}$ animals by caerulein treatment as described above.
b. Haematoxylin and eosin staining of pancreas sections at D3 and D8 of AP**.**
c. Representative images of α-Sma immunofluorescence, CD3 immunohistochemistry at D3 of AP and SHG signal at D8 of AP.
d. Quantification of α-Sma+ area/field, analysed using ImageJ.
e. Quantification of CD3+ area/field, analysed using ImageJ.
f. Quantification of SHG signal intensity. All images were acquired using 20x magnification. Scale bars correspond to 50µm.
Data is presented as Mean +/- SEM; N=5. Statistical analysis was performed using an unpaired t test with Welsh's correction; *P<0.05, ***P<0.001.

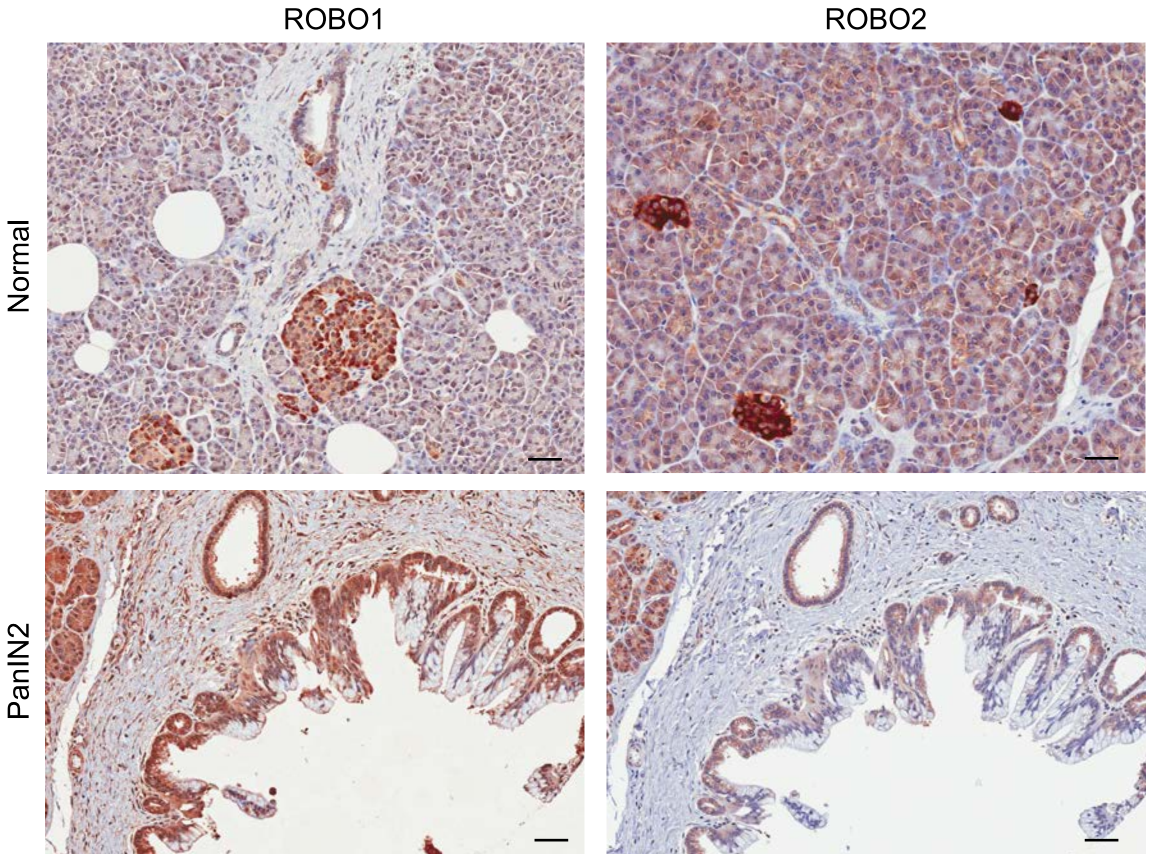

Supplementary Figure 13. ROBO1 and ROBO2 expression in human non-malignant pancreas
ROBO1 and ROBO2 immunohistochemistry (IHC) in normal human tissue and pre-malignant PanIN2 neoplastic lesions. Images are representative from n≥5 independent samples. Images were acquired using Aperio ImageScope software at a magnification of 20x. Scale bars correspond to 50µm.

SUPPLEMENTARY TABLES

PCR Array Catalog:	Mouse Cancer Inflammation and Immunity Crosstalk RT2 Profiler		
Test Group:	Robo2F/F		
Control Group:	Pdx1Cre		
Fold Difference:	1.5		
p-value:	0.05		
Genes Over-Expressed in			
Robo2F/F vs. Pdx1Cre			
Position	Gene Symbol	Fold Regulation	p-value
5	Ccl2	1.9534	0.327795
6	Ccl20	1.6259	0.140531
**15**	**Ccr5**	**1.797**	**0.001677**
22	Ctla4	2.1764	0.269506
28	Cxcl5	1.6451	0.308009
29	Cxcl9	1.5983	0.611151
**31**	**Cxcr2**	**12.719**	**0.014165**
33	Cxcr4	1.9313	0.07685
37	Fasl	3.077	0.115006
46	Ifng	1.9884	0.764896
65	Mif	6.7962	0.976416
76	Tlr2	3.9291	0.210226
**79**	**Tlr7**	**2.7628**	**0.044131**
**83**	**Trp53**	**1.6398**	**0.012093**
Genes Under-Expressed in			
Robo2F/F vs. Pdx1Cre			
Position	Gene Symbol	Fold Regulation	p-value
2	Aicda	-1.785	0.205666
13	Ccr2	-2.1805	0.924605
16	Ccr7	-2.1538	0.072451
21	Csf3	-2.252	0.187307
30	Cxcr1	-2.607	0.171788
32	Cxcr3	-1.7102	0.304525
34	Cxcr5	-1.5677	0.211357
35	Egf	-2.4531	0.135299
38	Foxp3	-6.1582	0.104892
41	Gzmb	-1.9698	0.151858
45	Ido1	-1.5843	0.431069
49	Il12a	-2.1416	0.109252
51	Il13	-2.1063	0.266497
53	Il17a	-2.4357	0.280037
**54**	**Il1a**	**-2.9762**	**0.014669**
61	Il5	-1.5683	0.543296
67	Myd88	-2.4876	0.86878
71	Ptgs2	-1.6465	0.238116

Supplementary Table 1. Complete list of genes analyzed using the RT2 Profiler PCR array Mouse Cancer Inflammation & Immunity Crosstalk (Qiagen) used to generate the Volcano Plot in Figure 5f.

Term	ID	Input number	Background number	P-Value	Corrected P-Value
**Focal adhesion**	**hsa04510**	**29**	**203**	**8.697E-16**	**2.551E-14**
**ECM-receptor interaction**	**hsa04512**	**18**	**82**	**4.532E-13**	**1.029E-11**
Protein digestion and absorption	hsa04974	18	90	1.791E-12	3.821E-11
**Wnt signaling pathway**	**hsa04310**	**18**	**143**	**1.577E-09**	**2.401E-08**
**PI3K-Akt signaling pathway**	**hsa04151**	**26**	**342**	**9.383E-09**	**1.271E-07**
**Cell adhesion molecules**	**hsa04514**	**16**	**146**	**7.395E-08**	**8.868E-07**
**Pathways in cancer**	**hsa05200**	**26**	**397**	**1.514E-07**	**1.725E-06**
cGMP-PKG signaling pathway	hsa04022	14	167	8.707E-06	7.412E-05
Malaria	hsa05144	8	49	1.045E-05	8.754E-05
**Axon guidance**	**hsa04360**	**14**	**176**	**1.511E-05**	**1.229E-04**
Proteoglycans in cancer	hsa05205	15	205	1.906E-05	1.517E-04
Vascular smooth muscle contraction	hsa04270	11	120	3.713E-05	2.724E-04
AGE-RAGE signaling pathway in diabetic complications	hsa04933	10	101	4.570E-05	3.291E-04
HTLV-I infection	hsa05166	16	259	6.965E-05	4.842E-04
Glycosaminoglycan biosynthesis	hsa00532	5	20	8.700E-05	5.895E-04
Arrhythmogenic right ventricular cardiomyopathy	hsa05412	8	74	1.497E-04	9.556E-04
Basal cell carcinoma	hsa05217	7	55	1.546E-04	9.851E-04
**MAPK signaling pathway**	**hsa04010**	**15**	**255**	**1.924E-04**	**1.191E-03**
Amoebiasis	hsa05146	9	100	2.121E-04	1.302E-03
Hippo signaling pathway	hsa04390	11	154	2.915E-04	1.726E-03
Hypertrophic cardiomyopathy	hsa05410	8	83	3.084E-04	1.795E-03
MicroRNAs in cancer	hsa05206	16	299	3.274E-04	1.894E-03
**TGF-beta signaling pathway**	**hsa04350**	**8**	**84**	**3.323E-04**	**1.919E-03**
**Regulation of actin cytoskeleton**	**hsa04810**	**13**	**215**	**3.933E-04**	**2.213E-03**
Hedgehog signaling pathway	hsa04340	6	47	4.506E-04	2.492E-03

Supplementary Table 2. Gene Set Enrichment analysis of genes whose expression significantly correlates with ROBO1, analysed using KOBAS 3.0 software to test statistical enrichment of KEGG pathway.

SUPPLEMENTARY METHODS

RNA *In Situ* hybridisation

Paraffin-embedded pancreatic tissue sections were incubated at 60°C for 1 hour followed by deparaffinization in Xylene (VWR, Amsterdam, NL – 28975.325) and dehydrated in 99% ethanol (VWR, Amsterdam, NL – 20821.330). Endogenous peroxidase was blocked with hydrogen peroxide (Advanced Cell Diagnostics Inc, Hayward, CA – 322330) for 10 min at room temperature (RT) followed by 2 washes in milliQ water (Millipore, Billerica, MA). Heat-induced epitope retrieval was performed for 15min at 100°C using RNAscope® Target retrieval (Advanced Cell Diagnostics Inc, Hayward, CA - 322000) followed by 2 milliQ washes and 99% ethanol rinse. Slides were dried at RT and hydrophobic barriers applied. Samples were incubated with RNAscope® protease plus (Advanced Cell Diagnostics Inc, Hayward, CA – 322330) at 40°C (singleplex – 30min, dual ISH-IHC – 1/5 dilution 15min) and subsequently washed with milliQ water. Samples were incubated with their respective probe for 2 hours at 40°C and afterwards washed for 2 min in RNAscope® wash buffer (Advanced Cell Diagnostics Inc, Hayward, CA - 310099). Amplification rounds 1-6 alternated from 30 to 15 min. After each amplification round samples were washed with RNAscope® wash buffer. Signal detection was performed by incubating samples with Alkaline phosphatase (solution of RNAscope® Fast A and B) for 10min and subsequently washed with milliQ water. After RISH, samples were counterstained with haematoxylin (Sigma, St Louis, MO – GHS1), dried at 60°C for 30min and mounted using VectaMount® (Vector Laboratories, Burlingame, CA - H5000).

RISH/Immunofluorescence multiplexing

After standard ISH procedure, tissue sections were washed in phosphate buffered saline supplemented with 0,1% tween (Sigma, St Louis, MO) (PBS-T) and endogenous peroxidase was blocked with protein block -serum free ready-to-use (X0909 - Dako, Glostrup, Denmark) for 10min at RT. After 3x5min washes, tissue sections were incubated overnight at 4°C with primary antibodies: anti-mouse E-cadherin (1/50, BD610181 – BD Pharmingen, San Diego, CA), anti-mouse α-smooth muscle actin (1/50, M0851 - Dako, Glostrup, Denmark), anti-mouse Amylase (1/100, SC46657 - Santa Cruz, Dallas, TX), anti-rabbit Vimentin (1/500, ab92547 – Abcam, Cambridge, UK), anti-rabbit CD3 (1/50, A0452 - Dako, Glostrup, Denmark). Rabbit (Vector Laboratories, Burlingame, CA - I-1000) and Mouse (Vector Laboratories, Burlingame, CA - I-2000) Isotype controls were applied to control slides. Antibodies were diluted in Dako antibody diluent (S0809 - Dako, Glostrup, Denmark). Next day samples were washed with PBS-T. Fluorophore-coupled secondary antibodies: alexa fluor® 488 anti-mouse, 647 anti-rabbit and 647 anti-mouse (all Jackson ImmunoResearch Inc, West Grove, PA) were incubated on their respective tissue sections for 1h at RT and subsequently washed with PBS-T. Tissue sections were coverslipped with Vectashield mounting medium containing Dapi (Vector Laboratories, Burlingame, CA - H1200).

Image analysis of Tgf-β/E-cad and Krt19/Amy colocalization

Nikon Imaging

Whole-section images were captured at 10-fold magnification (NA: 0.45) as a stack with a monochromatic image for each channel using a Nikon Eclipse TE2000-E microscope equipped with a Marzhauser Tango stage for large image microscopy with a fixed exposure time of 30msec for amylase and 80msec for Krt19/Amy. Individual 10X image fields where automatically stitched with 5% overlay, resulting in a whole-section 16-bit image.

Evos imaging

Whole-section images were captured at 20-fold magnification with fixed exposure times by Evos FL1 auto digital microscope. Automated image stitching resulted in a 16-bit image for image processing.

Morphometry (ImageJ)

Image processing was performed with Fiji, an open-source distribution of ImageJ (version 1.48d), developed at the National Institutes of Health, USA and allowing user extensibility via Java plugins.

In a first step, an ImageJ macro was created to automatically select the Dapi+ area on each section as a representation of total tissue region of interest (ROI).

Detection of colocalization or Tgf-β occupied E-cad area:

The manually verified tissue boundary ROIs were applied to the amylase and CK19 or E-cad and Tgf-β area image for automatic or investigator-validated area quantification. Within the boundary ROI, Fiji automatic or manually-set fixed thresholds were applied to select immune-stained signal. The fixed threshold was chosen as the median threshold that correlated most accurately with specific signal in several random ROI. Manual verification was performed by checking the conformity of each separate ROI. Image calculations and representations were performed with Imaris software.

Imaris analysis

Additional Image analysis was performed by introducing the images into Imaris version 9.1. (Bitplane software). Amylase, E-cad and Krt19 positive pixels were calculated as surfaces, whereas Tgf-β1 positive pixels were determined as spots. In order to determine colocalization we used the freeware Xtcoloc extension.

Recombination specific PCR for Robo2-flox animals

Specific primer sequences used for determining the presence and recombination of the Robo2-flox allele are detailed below:

Robo2flox allele:

Ro2-MEBAC15F	Forward	Intron 5 of Robo2	5 -CCAATCATAGTCTCTCCACG-3
Ro2-MEBAC15R	Reverse	Intron 5 of Robo2	5 -CCTCTGATTCAATGAGATGC-3

1,180-bp fragment from Robo2del5 allele:

Robo2koF	Forward	Intron 4 of Robo2	5-CCACTATGCTGGCTCTGTCTCACAC-3
Robo2R	Reverse	Intron 5 of Robo2	5 -GGTTTTGGAGGTCTTACTACGTAGC-3

1,390-bp fragment from Robo2 wild-type allele:

Robo2wtF	Forward	Intron 4 of Robo2	5 -CAACTTTTCCTTTTCCGGGAGG-3
Robo2R	Reverse	Intron 5 of Robo2	5 -GGTTTTGGAGGTCTTACTACGTAGC-3

RNA interference in Panc1 cells

Small interfering RNA (siRNA) gene expression knockdown studies were performed in Panc1 cells using the TriFECT RNAi kit (Integrated DNA Technologies, Leuven, Belgium). Each 27mer RNAi duplex was reverse transfected into Panc1 cells using Lipofectamine RNAiMAX (Life Technologies, Halle, Belgium) following the manufacturer's protocol. Final concentrations for each siRNA were 5nM. The following siRNA sequences were used: human Robo2 DsiRNA13.1 (5'-AGGAAUGGAUAGUGAAUCUAAAACG-3'), human Robo2 DsiRNA13.3 (5'-AGAAUCAAUUUUACCUGUCAUUGTA-3) and Negative Control DsiRNA that does not interact with any sequences in the human transcriptome.
At 96 h after transfection, cells were collected and RNA extractions were performed using Genelute mammalian total RNA miniprep (Sigma-Aldrich, Bornem, Belgium). RT–qPCR experiments were conducted as described above. Results are shown as the relative fold

expression compared to respective control treatment. RNA expression data normalization was carried out against the geometric mean of the three reference genes: SDHA, TBP and HPRT1.

Fluorescence Recovery After Photobleach

Pdx1^{Cre}; Robo2$^{F/F}$ mice were crossed with a previously described E-cadherin-GFP mouse line (Erami et al., Cell Reports 2016) to generate Pdx1^{Cre}; E-cadherin^{GFP}; Robo2$^{F/F}$ animals. After induction of acute pancreatitis, as described above, mice were euthanized in line with animal ethics guidelines. Freshly excised pancreas was placed into a glass bottom dish (Cellvis) in pre-warmed DMEM (phenol-red-free, Life Technologies) + 1g/l BSA (fatty acid free, Sigma-Aldrich) + 2mM L-Glutamine (Life Technologies) + 10mM HEPES (Sigma-Aldrich) + 50U/ml Penicillin/Streptomycin (Life Technologies). The tissue was maintained at 37°C and 5% CO_2. Fluorescence Recovery After Photobleach (FRAP) imaging was performed on an inverted Leica SP8 microscope at 488 nm excitation using the following settings: pixel dwell time 4 µs/px, pixel resolution 448x448. For FRAP imaging, after 5 pre-bleach frames a circular area with 2.5µm diameter (FRAP imaging) was bleached to approximately 50% of the original pre-bleach intensity using 405 nm excitation for one frame (Eram et al., 2016). After bleaching, images were captured every 1.6 s for ~ 7 minutes.

Recovery curves obtained from fluorescent intensity measurements were exported for exponential curve fitting, as described previously. Imaging data was corrected for drift using the phase correlation method and movement of junctions using an optical flow based approach as previously described[1]. Data were fitted using the following exponential function:

$$y(t) = y_0 + a * (1 - e^{-b*x})$$

The immobile fraction (F_i) was calculated as follows using values derived from the curve fit:

$$F_i = 100 * (1 - \frac{a}{t - y_0})$$

SUPPLEMENTARY REFERENCES

1. Erami Z*, et al.* Intravital FRAP Imaging using an E-cadherin-GFP Mouse Reveals Disease- and Drug-Dependent Dynamic Regulation of Cell-Cell Junctions in Live Tissue. *Cell Rep* **14**, 152-167 (2016).
